# Supplementary material for: Early recovery of leukocyte subsets is associated with favorable progression-free survival in patients with inoperable stage II/III NSCLC after multimodal treatment: a prospective explorative study
Source: Radiat Oncol. 2025 Mar 20;20:43. doi: 10.1186/s13014-025-02620-z (PMC11927295; doi:10.1186/s13014-025-02620-z)
Supplement: Supplementary file 1 — Supplementary Material 1 [file 13014_2025_2620_MOESM1_ESM.docx]

Supplement Tables and Figures

Table S1 Patient’s radiotherapy treatment characteristics

Patients Tumor Radiotherapy (based on PVT) Chemotherapy

PTV / ENIV PT (Gy) LN (Gy) ENIV (Gy)

(ml) tD / sD tD / sD tD / sD

B-001 91.5 / 1013.7 63.00 / 2.10 60.0 0/ 2.00 51.00 / 1.70 CiP / Vino

B-002 419.8 / 1102.7 63.00 / 2.10 63.00 / 2.10 51.00 / 1.70 CiP / Ptx

B-003 281.5 / 581.2 63.00 / 2.10 63.00 / 2.10 51.00 / 1.70 CbP / Vino

B-004 114.5 / 889.2 60.20 / 2.15 56.00 / 2.00 47.60 / 1.70 CiP / Ptx

B-005 401.4 / 1189.2 63.60 / 2.12 60.00 / 2.00 51.00 / 1.70 CiP / Vino

B-006 296.9 / 561.4 48.00 / 3.00 ––– 40.00 / 2.50 –––

B-007 59.0 / 433.6 63.60 / 2.12 60.00 / 2.00 51.00 / 1.70 CiP / Vino

B-009 166.4 / 533.9 63.60 / 2.12 60.00 / 2.00 51.00 / 1.70 CbP / Ptx

B-010 196.4 / 585.4 63.60 / 2.12 60.00 / 2.00 51.00 / 1.70 CiP / Vino

B-011 95.4 / 895.5 63.60 / 2.12 60.00 / 2.00 51.00 / 1.70 CbP / Vino

B-012 14.1 / 485.6 48.00 / 3.00 ––– 40.00 / 2.50 –––

B-013 563.9 / 1217.5 63.00 / 2.10 60.0 0/ 2.00 51.00 / 1.70 CiP / Ptx

B-014 82.9 / 622.3 61.60 / 2.20 61.60 / 2.20 50.40 / 1.80 CiP / Vino

B-016 27.2 / 867.4 63.60 / 2.12 60.00 / 2.00 51.00 / 1.70 CiP / Vino

B-017 43.9 / 494.8 64.48 / 2.08 62.00 / 2.00 52.70 / 1.70 CiP / Ptx

B-019 135.6 / 495.8 63.60 / 2.12 60.00 / 2.00 51.00 / 1.70 CbP / Vino

B-021 191.8 / 620.8 63.60 / 2.12 60.00 / 2.00 51.00 / 1.70 CiP / Ptx

B-022 120.9 / 437.4 63.60 / 2.12 60.00 / 2.00 51.00 / 1.70 CiP / Vino

B-023 1444.8 / 2293.2 63.60 / 2.12 60.00 / 2.00 51.00 / 1.70 CiP / Vino

B-024 116.4 / 683.0 63.60 / 2.12 60.00 / 2.00 51.00 / 1.70 CiP / Vino

Planning tumor volume (PTV), (elective nodal irradiation volume ENIV), lymph nodes metastases (LN), single integrated boost (SIB),

Total dose (tD), single dose per fraction (sD), organ at risk (OAR)

Carboplatin (CbP), Cisplatin (CiP), Pemetrexed (Ptx), Vinorelbin (Vino)

Tab. S2 Specifications of antibodies used for identification of leukocyte subpopulations

**Antibody Staining Panel (surface marker)**

Marker FL Clone Conc. Vendor

CD3 PerCP-Cy5.5 SK7 1:33 eBioscience

CD4 APC-H7 RPA-T4 1:33 BD

CD8 BV496 RPA-T8 1:50 BD

CD14 BV421 M5E2 1:40 BD

CD16 PE-CF594 3G8 1:67 BD

CD19 A700 HIB19 1:50 BioLegend

CD20 A700 2H7 1:50 BioLegend

CD56 APC N901 (NKH-1) 1:20 Coulter

in 50 µl Brilliant Staining Buffer (BD)

**Tab. S3** Medians of absolute cell counts (10^9^ cells / L) of leukocytes at time points RTend, C.1, C.2, and C.3 within the favorable PFS group (PFS ≥12 months). Median; RTend n=8, C.1, C.2, C.3 n=9; Student’s t-test, * p<0.05, ** p<0.01.

ALC PFS ≥12 months 10^9^ cells / L p

RTend C.1 C.2 C.3 RTend - C.1 RTend - C.2 RTend - C.3 C.1 - C.2 C.1 - C.3 C.2 - C.3

Lymphocytes 0.451 1.180 1.980 1.010 0.0294* 0.0073** 0.0130* 0.2089 0.1588 0.3680

total CD3+ T cells 0.449 1.080 1.450 0.786 0.0411* 0.0109* 0.0314* 0.2303 0.2616 0.4904

CD4+ T cells 0.236 0.309 0.524 0.516 0.2300 0.1094 0.0877 0.2329 0.2290 0.4554

CD8+ T cells 0.116 0.290 0.799 0.427 0.0160* 0.0039** 0.0192* 0.3073 0.2473 0.3776

B cells 0.005 0.006 0.071 0.071 0.1301 0.0107* 0.0085** 0.0113* 0.0078** 0.2422

NK cells 0.048 0.261 0.174 0.213 0.0660 0.0846 0.0110* 0.3462 0.2932 0.1415

Eosinophils 0.080 0.100 0.190 0.011 0.2714 0.0247* 0.2902 0.1019 0.4487 0.0556

Neutrophils 2.240 3.700 4.710 5.650 0.2413 0.0442* 0.0164* 0.0521 0.0152* 0.1217

**Tab. S4** Spearman correlation analysis between PFS categories (PFS < 6 months, ≥6 <12 months, ≥12 months) and AUC of blood cell populations using Spearman’s correlation test (* p<0.05, ** p<0.01, *** p<0.001).

| AUC | p |
| --- | --- |
| Lymphocytes | 0.040* |
| total CD3+ T cells | 0.011* |
| CD4+ T cells | 0.001*** |
| CD8+ T cells | 0.096 |
| B cells | 0.221 |
| NK cells | 0.0002*** |
| Eosinophils | 0.297 |
| Neutrophils | 0.638 |
| NLR | 0.226 |

**Tab. S5** Plasma IL-6 concentrations (pg/ml) for the patients before treatment begin (A.1) and during recovery phase (RTend-C.3) (red: PFS < 6 months, grey: ≥6 <12 months, green: ≥12 months).

| **IL-6 (pg/ml)** |  |  |  |  |  |  |  |
| --- | --- | --- | --- | --- | --- | --- | --- |
|  | PFS group | A.1 | RTend | C.1 | C.2 | C.3 | SD RTend-C.3 |
| B-001 | <6 | 33,3 | 11,6 | 11,5 | 51,8 | 108 | 45,65 |
| B-002 | <6 | 22,4 | 73,4 | 17,9 | 21,1 |  | 31,16 |
| B-003 | <6 | 647 | 521 | 669 | 319 |  | 175,69 |
| B-004 | >12 | 4,2 | 8,7 | 6,1 | 3,1 | 4,2 | 2,45 |
| B-005 | ≥6>12 | 15,8 | 25 | 6,2 | 3,6 | 13,6 | 9,59 |
| B-006 | ≥6>12 | 11,5 |  | 30,3 | 40,5 | 27,3 | 6,92 |
| B-009 | >12 | 9,7 | 8,2 | 13,7 | 9,1 | 2,5 | 4,60 |
| B-010 | <6 | 9,8 | 6,5 | 51,7 | 22,4 |  | 22,93 |
| B-011 | ≥6>12 | 28,4 | 24,3 | 8,8 | 18,7 | 5,2 | 8,80 |
| B-012 | ≥6>12 | 4,1 | 22,5 | 28,9 | 5,2 | 4,5 | 12,32 |
| B-013 | <6 | 8,2 | 6,5 | 5,2 | 16,5 | 6,6 | 5,24 |
| B-014 | ≥12 | 5,2 |  | 4 | 3,4 | 2,6 | 0,70 |
| B-016 | ≥12 | 9,5 | 8,2 | 4,1 | 5,2 | 7,2 | 1,86 |
| B-017 | ≥12 | 6,1 | 18,6 | 3,7 | 7,2 | 6,1 | 6,63 |
| B-019 | ≥12 | 2,7 |  | 3,3 | 1,5 | 1,7 | 0,99 |
| B-021 | ≥6>12 | 18,4 | 10,1 | 1,5 | 3,3 | 1,7 | 4,05 |
| B-022 | ≥12 | 2 | 8,2 | 4,9 | 3 | 2,9 | 2,48 |
| B-023 | ≥12 | 60,1 | 21,5 | 16,4 | 11,3 | 6,7 | 6,39 |
| B-024 | ≥12 | 50,3 | 19,2 | 14,1 |  | 19,9 | 3,17 |

**Figure S1**

**Patient characteristics, study design, data collection**

20 patients were included in this trial. Two patients received RT alone, 18 patients were treated with platinum-based concurrent cCRT, and 7 patients received additional ICI either concurrently (nivolumab, 480 mg every 3 weeks up to one y, including induction therapy with nivolumab prior to and during cCRT) or sequentially (durvalumab, 10 mg per kg body weight every 2 weeks for up to 1 y) to cCRT. In all patients, RT was applied with a median cumulative dose in equivalent 2 Gy fractions (EQD2) of 64 Gy (range 52-65 Gy). Two patients treated with cCRT received pembrolizumab salvage therapy after time points C.1 and C.2, respectively. Thus, subsequent time points of these patients were excluded from the analysis. One RT alone patient received a tyrosine kinase inhibitor between C.4 and C.5 for 8 weeks.

During treatment, 6 patients developed distant metastases (DM) while 9 had local recurrences (LR). Seven patients died during follow-up (within 1 y after the end of RT), 18 patients survived at least 6 months (mo), 14 patients at least 12 months.

Blood was drawn on different time points before, during and after treatment up to 1y follow up, indicated below in the timeline. For seven patients, blood of all time points could be collected. Others either missed a time point, revoked consent, or suffered from fatal disease progression. If not stated otherwise, B-010 and B-013 were excluded from analysis examining impact of ICI-treatment because they received salvage therapy using pembrolizumab.

**
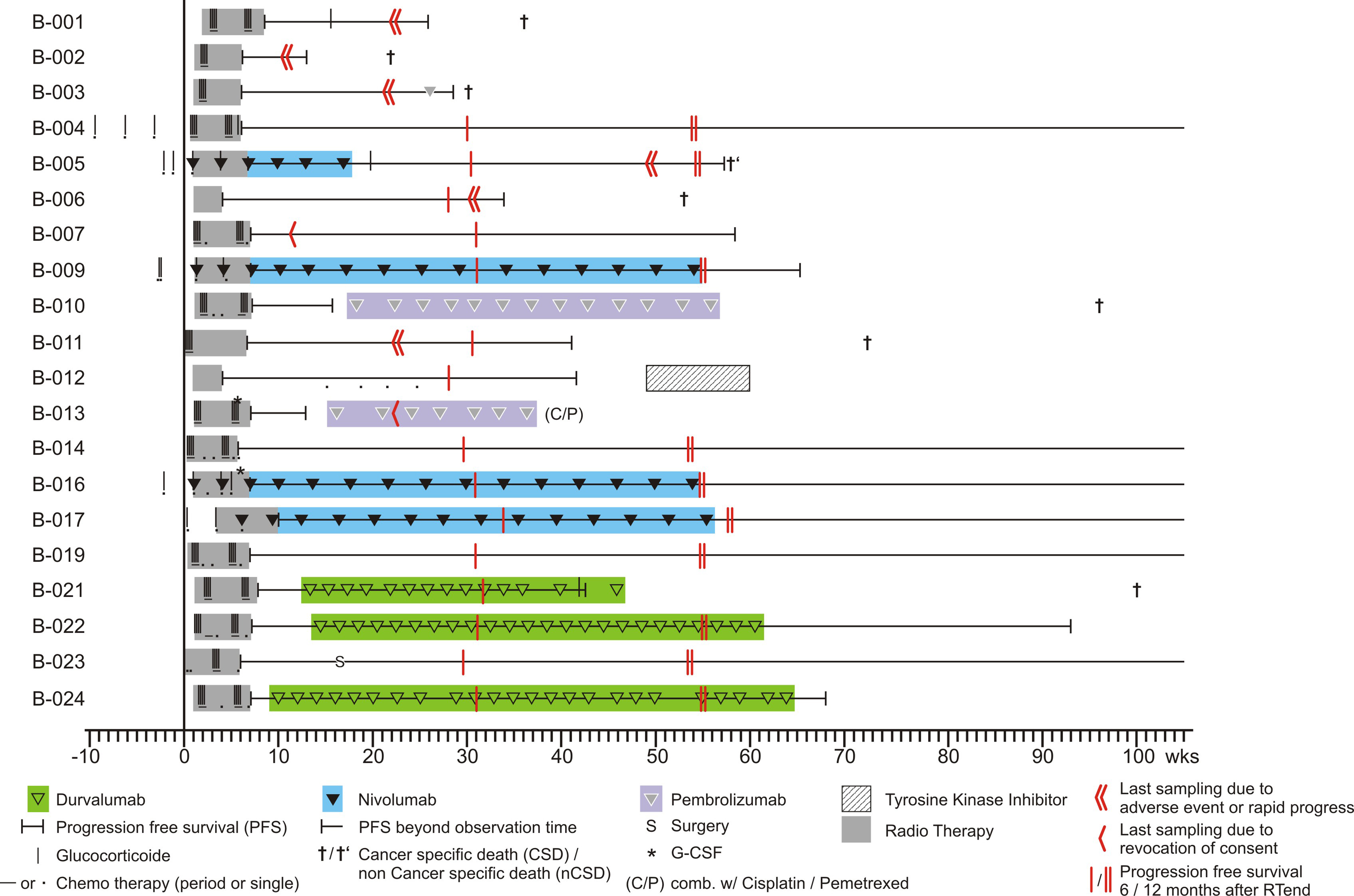
**

**Fig. S1** Summary view showing 20 patients (vertically on the left), the timeline of treatment, medication, progression free survival, and overall survival (horizontally to the right).

Figure S2

**Sample Preparation and Gating Strategy**

Blood was collected using 3K-EDTA S-Monovette tubes (Sarstedt, Germany) and samples were analyzed within 3 hours. Using 100 µl of whole blood, erythrocytes were lysed using Q-Prep Workstation (Coulter, Germany). Cells were washed in 1 ml and resuspended in 100 µl FCM-Buffer (PBS without Ca^2+^ and Mg^2+^, supplemented with 2 mM EDTA and 2% FCS). For immuno-phenotyping, blood cells were stained on ice in the dark with a surface marker antibody panel to assess main blood cell subpopulations (Tab. S2). Cells were suspended in 250 µl FCM-Buffer, 50 µl (half the volume of blood) Flow-Count Fluorospheres (Beckman Coulter, Germany) were added for absolute cell count determination and analyzed on a Beckton Dickinson LSR II flow cytometer with Diva Software (version 8). A compensation matrix was generated using BD CompBeads, for flow data analysis FlowJo Software (version 10) was used. The staining protocols were established prior to the experiments, the formula for calculating the absolute cell numbers was (specified count of Flow-Count beads (/µl) * cell count in assay) / (count of Flow-Count beads in assay * 2).

To identify immunocyte subpopulations, leukocytes were gated within FSC-A vs. SSC-A to exclude debris. T-cells were defined as CD3+ CD14- CD16- CD19/20- single cells (FSC-A vs. FSC-H) and subsequently subdivided into CD4+ and CD8+ T-cell populations (Fig. S2). B-cells were identified as CD19/20+ CD14- CD56- CD16- single cells.

To gate for NK-cells, we excluded highly CD16 positive neutrophils in the CD16 vs CD3 pre-gate. Then CD3+, CD19+20+ and CD14+ cells were excluded, and the remaining single cells cell population was gated for CD56+ CD16+. We excluded highly CD16 positive neutrophils at an early stage for increased clarity and overview. In our flow data analysis, CD16-expression of blood cell subsets other than neutrophils, including the NK-cells or CD16+ monocytes, were in each sample consistently well-separable. In case CD3-positive events were included in the pre-gate, they are excluded in the subsequent gate of the CD16 vs CD56 dot plot as far as these events are not CD56+ and CD3+. Latter cell population accounts for max. 20% of the CD3-positive cells which, for this analysis, has a minimal potential to alter defining the NK-cell population and the cell counts.

We noted a high autofluorescence spill of the neutrophil granulocytes into the emission channel used to identify CD3+ T-cells (ex 488 nm, em 695/40 nm) (Fig. S2). Neutrophils were identified by their high SSC and high expression of CD16 (not shown). CD3+ T-cells remained well definable within the SSC vs. CD3-PerCP-Cy5.5 dot plot. In the experiments shown, no live dead stain was used. Blood was analyzed within 1-2 hours after withdrawal, not expecting significant quantities of dead cells. Furthermore, additional experiments confirmed minimal amount of dead cells and viability of CD3+ T-cells.

FlowCount (Beckman Coulter) counting beads were gated within FSC-A vs. SSC-A and subsequent event count was determined within 695nm emission channel (PerCP-Cy5.5) vs. SSC-H dot plot.

**
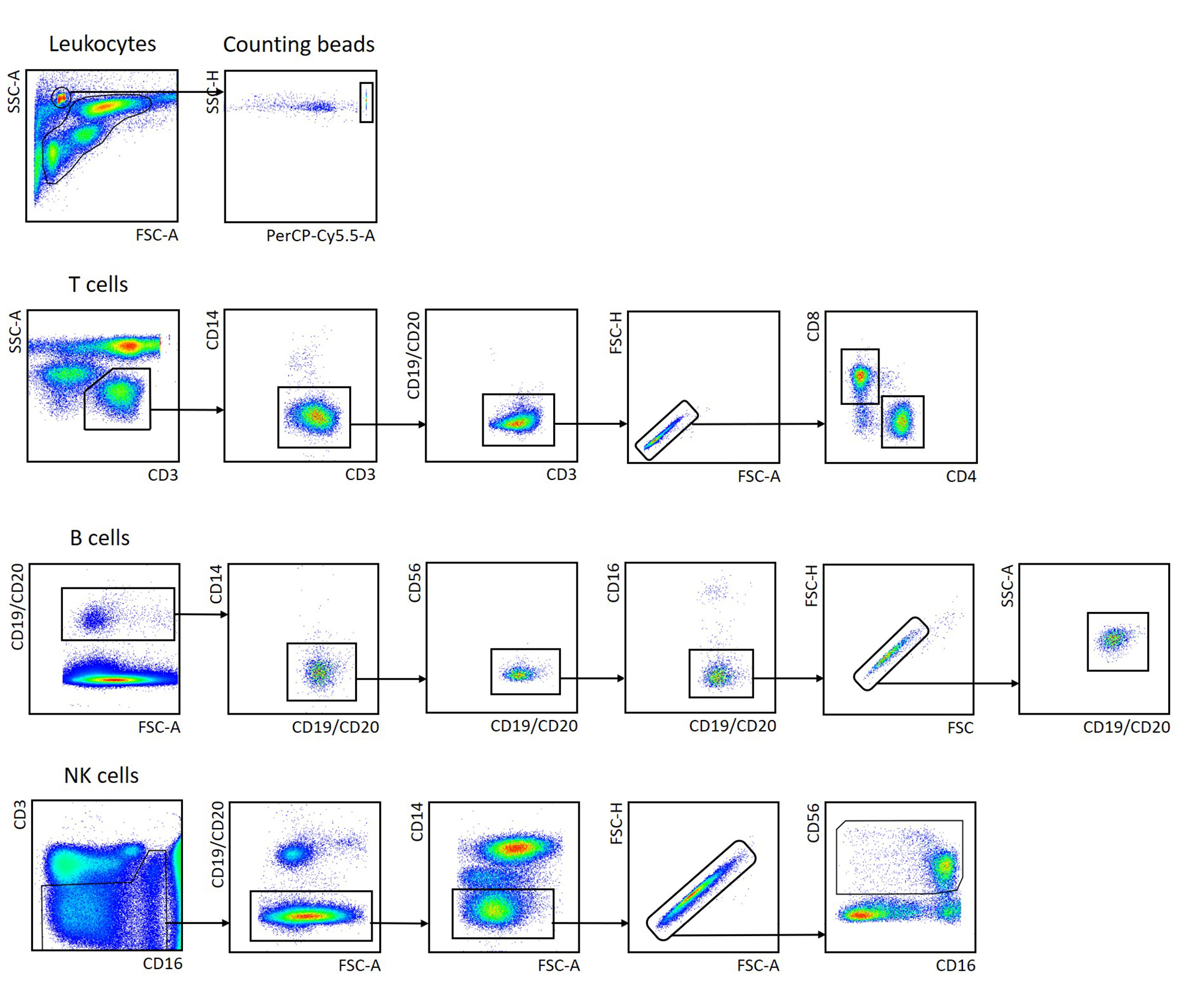
**

**Fig. S2** Gating strategy for the determination of the different cell population in peripheral venous blood. In each run counting beads were included as internal standard to calculate absolute cell counts.

**Figure S3**

**Progression Free & Overall Survival of patients with high, medium and low (UQR, IQR, LQR) lymphocyte cell counts at time point A.1 or C.3 as well as treatment groups**

Absolute lymphocyte counts (ALC) at A.1 trend to predict OS (b) but not PFS (a) (positive correlation). At time point C.3 (6 months post-RT/cCRT) higher ALC trend to predict a longer OS (d). Looking at treatment groups (e, f) addition of ICI is beneficial for PFS and OS.

**
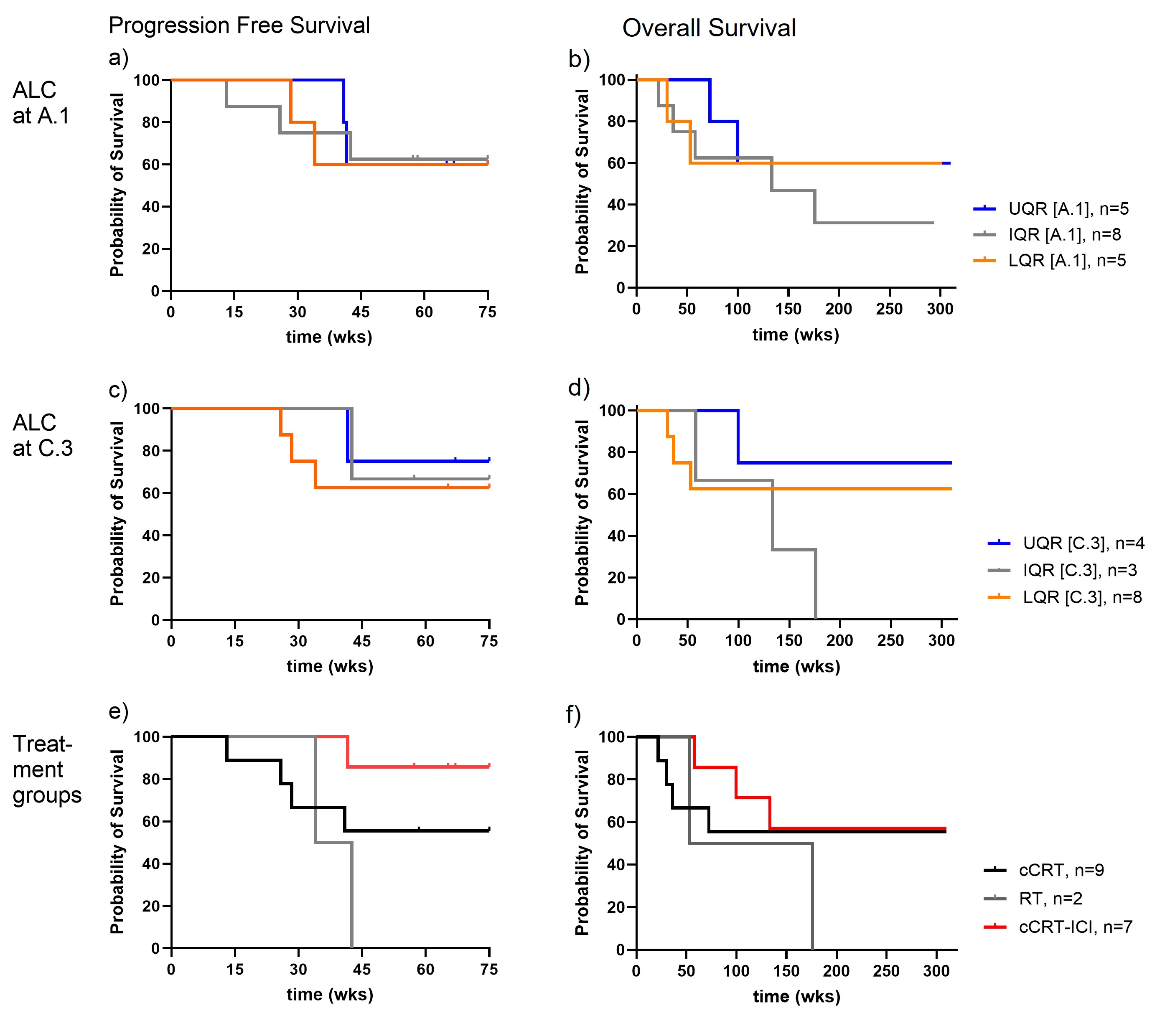
**

**Fig. S3** Survival Analysis (PFS, OS) of patients grouped according to the LQR, IQR, and UPR of the ALC at time point A.1 (a,b), and C.3 (c,d) as well as treatment group (e,f). For statistical analysis Logrank test (Mantel Cox) was performed, all not significant. Numbers of total patients at risk see legend.

**Figure S4**

**Dynamics of leukocyte subpopulations after RT**

Exploring the predictive value of absolute count (ALC) in lymphocyte subpopulations at the time point RTend and time points during follow-up revealed that the favorable PFS group (≥12 months) showed significant increase in AC from RTend to C.2 (3 mo after treatment end) for B cells, eosinophils, and neutrophils. From RTend-C.3 (6 months after treatment end) B cells, NK cells and neutrophils were significant (p>0.05).

**
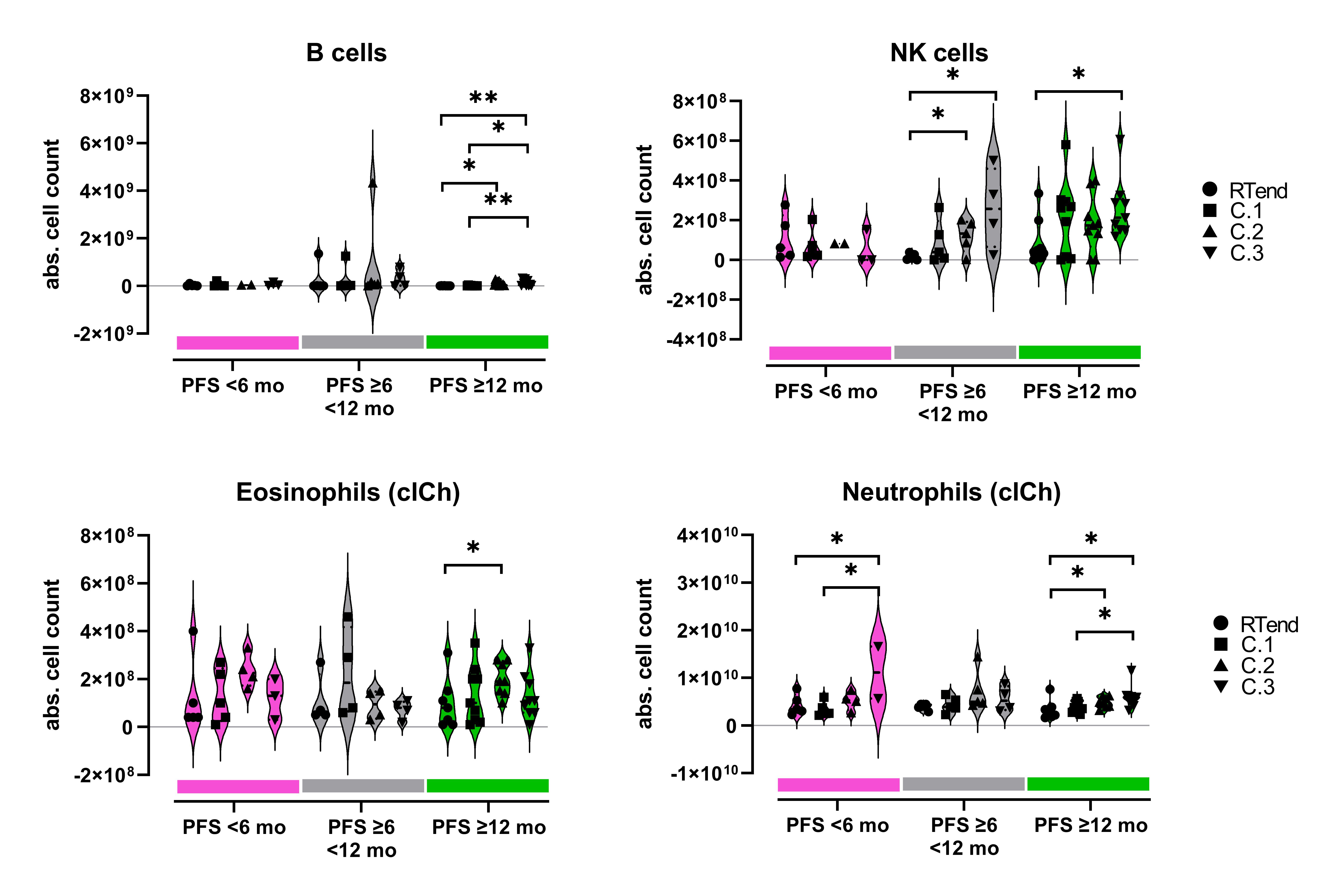
**

**Fig. S4** Absolute counts of lymphocytes and lymphocyte subpopulations at the time points RTend, C.1, C.2, and C.3 for the PFS groups (red PFS<6 mo, gray PFS ≥6<12 months, green ≥12 months). Each dot represents an individual. Comparison was made between the abs. cell counts at the different time points within each PFS group. Cell counts were derived from flow cytometry (gating strategy in Fig. S2), neutrophil and eosinophil counts were determined by clinical chemistry routine lab (clCh). Bold dashed line in the violins indicate the median, the light dotted lines the lower and upper quartile. Student’s t test, * p<0.05, ** p<0.01.

**Figure S5**

**LDA using blood cell subpopulations separates patients according to PFS**

Partition plots of all predictor variable combinations are shown for a more detailed information on their discriminant contribution towards the PFS groups.

**

**

**Fig. S5** Partition plot of linear discriminant analysis including discrimination error rates of the different cell subtype combinations. AUC of cell counts of the interval from RTend-C.3 (6 months after RT) were used and Z-score normalized. 1 represents PFS < 6 months, 2 represents PFS ≥6<12 months, and 3 represents PFS ≥12moths.
